# Editorial: How to better understand and treat children and adolescents suffering from eating disorders

**DOI:** 10.3389/fpsyt.2023.1209371

**Published:** 2023-06-13

**Authors:** Fiona McNicholas, Josefina Castro-Fornieles, Dasha E. Nicholls, Ulrike M. E. Schulze

**Affiliations:** ^1^School of Medicine, University Dublin, Dublin, Ireland; ^2^Department of Child and Adolescent Psychiatry and Psychology, Hospital Clinic Barcelona, Barcelona, Spain; ^3^Centro de Investigación Biomédica en Red de Salud Mental (CIBERSAM), Madrid, Spain; ^4^Institut d'Investigacions Biomediques August Pi i Sunyer (IDIBAPS), Barcelona, Spain; ^5^Department of Medicine, Institute of Neuroscience, University of Barcelona, Barcelona, Spain; ^6^Imperial College London, London, United Kingdom; ^7^Department of Child and Adolescent Psychiatry and Psychotherapy, University of Ulm, Ulm, Germany

**Keywords:** eating disorders, children and adolescents, etiology, attachment, cultural aspects, transition, treatment

Eating disorders (ED) usually first appear between the ages of 13 and 25. They continue to be of clinical concern by virtue of increasing incidence in younger populations and in boys, earlier onset, and risk of chronicity.

The COVID-19 pandemic saw an increase in new and relapsing cases of ED, with surges of referrals to both pediatric and adult general hospitals and to community services (1). Despite significant advances in psychological and pharmacological therapies for many mental illnesses in recent years, and development of manualized protocols mainly for anorexia nervosa (AN) and bulimia nervosa (BN) among youth, outcomes for individuals with ED remain unsatisfactory for many (2). Currently, medical and psychological adverse sequelae continue to contribute to premature deaths and poor quality of life.

In this context, and to counterbalance potential therapeutic nihilism, our Research Topic addressing updated research in ED disorders is timely. Papers included cover biological, family and cultural components on individual illness development and trajectories.

Historically adverse family interactions were seen as significant contributing factors to the origins and maintenance of these disorders in adolescents, with systemic family therapy advocated as the treatment of choice. Subsequent clinical work and research has enlightened us to the complex bio-psycho-social nature of ED, genetically mediated and with significant personal, cognitive and social components. These areas, including the role of parents, culture and attachments, are further scrutinized in this issue.

An overall review highlighting the potential protective role of parental relationships is well described by Izydorczyk et al. in their paper. Attachment experiences during early childhood provide an essential basis for future resilience, including self-acceptance and confidence, with both positive and potentially adverse effects.

Starting from here, Izydorczyk et al. dare to suggest an approach that promotes understanding between the child's perception of parental bonding, sociocultural influences on appearance, body image and ED risk. Key messages here remind us of the perils of maternal overprotection and emphasizing the importance of paternal care. The import of socio-cultural influence is exposed, with the knowledge that these internalized standards, including lower clarity of self-concept and identity confusion, are formed early and are more often perceived as egosyntonic (Izydorczyk et al.), hence providing treatment opportunities.

Other aetiological factors are proposed by Verschueren et al. in their large longitudinal cohort of adolescents, mostly females. They identified cognitive emotional dysregulation—namely “rumination, catastrophizing, and less positive reappraisal”—as an indirect factor paving the way to development of an ED (Verschueren et al.). They proffer that the distressing key symptom of body image disturbance, the nature of the self-perception of the mirroring self, may emanate from a dissociation between the psycho-physiological reactivity and the subjective response to body exposure (Knejzlíková et al.). Another irritation-prone developmental step is the subjective search for identity (3). Here, too, the family and cultural context provides a novel framing. Taking this into account, and exemplary in Brazil, Ramalho et al. have intensively investigated inter-acting factors related to family identities, and the interplay between food and emotions across generational boundaries. Their call to consider recognize and support the autonomy of the young person reminds us of the vulnerable age of youth, and recognizes ethical conflicts surrounding transitional care, where a balance needs to be struck between paternalism and empowerment of youth and their decision making (4).

Given that ED onset typically occurs over a broad developmental period, it is important to ensure seamless transitional pathways exist between child and adult services. Prior transition specific research has highlighted that youth with ED have been found to be least likely to transition to adult service (5). The ego syntonic and averse to help nature of many EDs means that many young adults are unsupervised and lost to follow up when moving out of home, and for some with the most serious of outcomes. Such barriers have been well described by contributors Nadarajah et al. in their qualitative exploration of youth from pediatric to adult care. Potential improvements might come from better coordination and the use of “Transition Passports” (Nadarajah et al.). Transitional and stepped care approaches are also described in this issue and when linked with motivational work have been found effective (Heider et al.).

There has been a wealth of research examining the link between the gut-brain axis and the role of the microbiome in our general health and wellbeing and genesis of specific diseases.

In addition to genetic factors, the type and quantity of food eaten contributes to the diversity of the millions of micro-organisms lining our digestive track and changes in food consumption and preparation has been reflected in significant deviations in our microbial flora. Both beneficial and harmful microbes exist, inducing those with anti or pro inflammatory immune function, either promoting or hindering physical and mental health. The changed microbial flora among those with AN compared to healthy controls remind us of the perils regarding the limitations of conceptualizing AN as a purely psychiatric or “brain centric” condition (6). Ghenciulescu et al. argue for a broader recognition of a potential contributory role of the microbiome in the etiology and maintenance of ED. They highlight he potential adverse effects of standard nutritional rehabilitation and rapid refeeding, such as severe GI distress. Additionally, they suggest the development and possible therapeutic advantages of microbiome-based therapies for patients with AN, such as fecal microbiota transplants and probiotic adjunctive treatments.

Based on this collection of papers, we can be optimistic of continued developments in search of a clearer understanding of the origins of the various ED and optimizing treatment outcomes in this vulnerable group.

## Author contributions

FM and US created the first draft. JC-F and DN extended with additions and suggestions for corrections. All authors finally contributed and agreed on the final version.
